# Green Synthesis of Silver Nanoparticles for Preparation of Gelatin Films with Antimicrobial Activity

**DOI:** 10.3390/polym14173453

**Published:** 2022-08-24

**Authors:** Xóchitl A. Pérez-Marroquín, Gabriel Aguirre-Cruz, Gieraldin Campos-Lozada, Graciela Callejas-Quijada, Arely León-López, Rafael G. Campos-Montiel, Laura García-Hernández, Abraham Méndez-Albores, Alma Vázquez-Durán, Gabriel Aguirre-Álvarez

**Affiliations:** 1 Instituto de Ciencias Agropecuarias, Universidad Autónoma del Estado de Hidalgo, Av. Universidad Km. 1 Rancho Universitario, Tulancingo C.P. 43684, Hidalgo, Mexico; 2 Centro de Desarrollo en Nanotecnología, Universidad Tecnológica de Tulancingo, Área Electromecánica Industrial, Camino a Ahuehuetitla No. 301, Colonia Las Presas, Tulancingo C.P. 43642, Hidalgo, Mexico; 3 Uni-Collagen S.A. de C.V., Arnulfo González No. 203, El Paraíso, Tulancingo C.P. 43684, Hidalgo, Mexico; 4 Unidad de Investigación Multidisciplinaria L14-A1 (Ciencia y Tecnología de Materiales). Km 2.5 Carretera Cuautitlán-Teoloyucan San Sebastián Xhala, Cuautitlán Izcalli C.P. 54714, Estado de México, Mexico

**Keywords:** nanoparticle, *Thuja orientalis*, green technology, gelatin films, antimicrobial activity

## Abstract

Silver nanoparticles were successfully synthesized using *Thuja orientalis* aqueous extract and AgNO_3_ as a precursor. UV–Vis showed a distinct absorption peak at 424 nm attributed to silver nanoparticles due to their surface plasmon resonance. Atomic absorption analysis reflected an increase in the concentration of nanoparticles in relation to the progress of the synthesis, obtaining a peak concentration value of 15.7 mg/L at 50 min. The FTIR spectra revealed the characteristic functional groups of phytomolecules involved in the silver–ion binding process, such as R–O–H (3335 cm^−1)^ O=C–OH (2314 cm^−1^) and C—C=C (1450 cm^−1^). At 50 min, zeta potential showed the stability of the nanoparticles with the value of −21.73 mV. TEM micrographs revealed the formation of spherical nanoparticles with an average size of about 85.77 nm. Furthermore, films incorporated with nanoparticles exhibited a Tg from 66.42 °C to 73.71 °C and Tm at 103.31 °C. Films from the G22 formulation presented excellent antibacterial properties inhibiting the growth of *Staphylococcus aureus*. *Thuja orientalis* aqueous extract could be a low-cost, eco-friendly, and efficient reducing and capping agent for the synthesis of nanometric-sized Ag particles. Gelatin films with nanoparticles are expected to have high potential as an active food packaging system.

## 1. Introduction

Green nanotechnology represents a new challenge to researchers around the world to widen the horizon on the potential capacity of nature to eliminate or diminish the environmental risks caused by the use of inorganic pollutants and encourage the replacement of these materials with new environmentally friendly alternatives based on nanomaterial synthesis [1]. Nanomaterials engineered through conventional physicochemical routes have caused a great environmental footprint which has forced the evolution of synthesis methods to be green-oriented to provide nanoparticle synthesis with a minimal natural habitat damage cost. The use of plant-based extracts has been an alternative to consider because of their amino acids, flavonoids, aldehydes, ketones, amines, carboxylic acids, phenols, and protein components. These materials can provide electrons that function as reducing agents for the synthesis of nanoparticles. The use of reducing agents provides electrons to reduce the metallic ions. Through the addition of capping agents, nanoparticles can be stabilized, avoiding aggregation by imputing repulsive forces that control the growth of nanoparticles [2]. The exploration of different antioxidant constituents of plant extract has been conducted to control the size and shape of silver nanoparticles for cancer, antifungal, antibacterial, and antioxidant treatments [3,4]. However, for experimental works involving extract plants for the synthesis of silver nanoparticles, special considerations should be taken in the selection of plant constituents in order to estimate the environmental impact and life cycle of plants [5]. 

On the other hand, the size of silver particles can be regulated by choosing the appropriate concentration of metallic salts and varying the reaction temperature or time [6]. Most of the reducing agents are dangerous and toxic. For this reason, it is necessary to develop environmentally friendly, nontoxic reducing agents for novel applications in material science innovation [7]. *Thuja orientalis* belongs to a genre of the coniferous tree with perennial leaf located in the cypress Cupressaceae family. It is a good source of natural compounds with significant antioxidant and antimicrobial activity because of its high content of polyphenolic compounds [8]. In addition, it is distributed widely throughout China, Japan, and Korea [9]. The α-thujone oil extracted from the leaves of this plant can be used to treat affections of the skin, blood, gastrointestinal system, kidney, brain, and tumors. Previous studies have shown that the leaves of *Thuja orientalis* have antioxidant, anti-inflammatory, antiallergic, anticancer, and melanin-inhibitory effects [10]. In addition, they contain several components that could be used in the synthesis of nanoparticles, i.e., rhodoxanthin, amentoflavone, hinokiflavone, quercetin, myricetin, carotene, xanthophylls, and ascorbic acid [11]. Ærøe Hyllested et al. [12] reported the synthesis of silver nanoparticles (AgNPs) with sizes between 10 and 300 nm using extracts from pineapples and oranges as reducing agents. Although it is a novel alternative for the synthesis of nanoparticles, the availability of the extract is subject to the harvesting period of the fruit, limitinging the synthesis to certain months of the year. Umadevi et al. [13] and Prathna et al. [14] reported the synthesis of AgNPs with sizes of 10 nm and less than 50 nm using *Solanum lycopersicum* and *Citrus limon* extracts, respectively. However, these food materials may have potential applications in the cosmetic and food industry because of their excellent contributions to human health as antioxidants and antimicrobials [15,16]. Philip et al. [17] used a honey solution as a reductant of AgNO_3_ to obtain AgNPs with the size of 4 nm. This raw material could be better applied to other health applications because it possesses a wide spectrum of therapeutic properties such as anti-inflammatory, antibacterial, antimutagenic, antidiabetic, antiviral, anticancer, and antitumor [18]. Honey could play a more important role as a modulator of different types of diseases. Awad et al. [19] synthesized AgNPs by using orange peel extract as a reducing and stabilizing agent. The synthesis yielded nanoparticles with an average size of 91 nm. In another study, Vijayaraghavan et al. [20] reported nanoparticles of 87 nm with undefined shapes using the extract of *Trachyspermmum ammi*. The election of *Thuja orientalis* extract for the synthesis of AgNPs is due to its wide variety of active compounds that can help in the reduction in Ag ions. These extracts are economical, safe, nontoxic, and friendly to the environment [21].

It is well known that petrochemical-based plastics such as polystyrene and polyethylene are commonly used as plastic packaging materials and considered nonbiodegradable products and can pose environmental problems [22]. Fortunately, the application of nanotechnology has developed edible nanocoating materials oriented to the active food packaging industry. These biodegradable nanofood packaging materials have been prepared from polysaccharides (cellulose, starch, and chitosan) and protein (gelatin, soy, and casein). The performance of films prepared from these sources depends on the hierarchical structure and their semicrystalline nature [23]. Gelatin is a protein obtained from the denaturation of collagen, and it is one of the most used biopolymers for the manufacture of a range of products because of its outstanding filmogenic properties. Furthermore, it is produced at a low cost while offering its unique properties as an outer film to secure food from moisture loss and exposure to light and oxygen [24]. This protein has become one of the most suitable renewable raw materials for food packaging as it is a green alternative to combat the environmental impact of plastic waste [25]. It is also an excellent carrier of bioactive components, gelling, water binding properties, and a good microencapsulation agent to entrap functional components [26]. A major advantage of gelatin-based film packaging is its high biodegradation rate [27]. The molecular and functional properties of gelatin films depend on the extraction, purification, and processing treatments used to produce functional ingredients [24]. One of the drawbacks of gelatin films is their water solubility and viscosity. It is greatly affected by natural weather conditions and air humidity. Gelatin is compatible with several biopolymers (e.g., proteins and polysaccharides) to develop composite films with extended biological and technological functionality. The presence of polysaccharides can modify the protein surface structure and weaken the formation of the protein network structure by increasing protein hydrolysis. This blend improves the physicochemical and biological properties of the resulted films [28]. Gelatin films have been reported as a good carrier for metallic ions and possess a good matrix for these active agents to perform their specific functions for enhancing the safety, stability, antimicrobial, functionality, and shelf life of food products [24]. Gelatin is still one of the key food hydrocolloids for the food industry. The knowledge of gelatin structure is not fully understood, along with some functional properties. It includes the impact of the initial raw material properties on the performance of finished products, for example, films with AgNPs. Although the use of metal NPs as antimicrobials in food packaging is a grown-up technology, concerns about the risks associated with the intake of Ag ions migrated into food still exist. Consequently, the European Food Safety Agency [29] provides guidance regarding the upper limits of Ag migration from packaging, and suggestions are not to exceed 0.05 mg/kg of food. This means that evaluations of silver composites concerning Ag migration profiles are needed to guarantee antimicrobial effectiveness while observing current legislation. 

Recently, polymer-based films have attracted significant attention in food packing because of their excellent film-forming ability [30,31]. Gelatin-based films are water-soluble biopolymers composed of a triple helix with repeating glycine–proline–hydroxyproline units [31,32]. They show different characteristics when mixed with various natural extracts, additives, or nano reinforcements. The latter have excellent antibacterial activity, which not only ensures food safety against pathogens but also restricts the oxidation process and preserves food freshness for a long period of time [33]. However, there is a concern about the safety of silver in its application in packaging materials because of the migration rate of silver ions from the film matrix. This migration is limited to 0.05 mg Ag/kg of food, and more research is required to investigate its behavior [34].

Although many vegetal sources have been used to synthesize nanoparticles, less is known about the *Thuja orientalis* plant as a reducing/capping agent. Consequently, in this work, the synthesis of AgNPs at different times was investigated with the addition of this plant aqueous extract. Additionally, AgNAPs were applied to gelatin solutions for the preparation of films with antimicrobial activity.

## 2. Materials and Methods

### 2.1. Plant Source

Fresh leaves of *Thuja orientalis* with no evidence of plague infestation or illnesses were used. Leaves were washed with deionized water to remove loosely adhering particles and water-soluble impurities and cut into thin pieces ignoring the stems.

### 2.2. Preparation of Plant Extract

Finely chopped leaves were weighed (1.5 g), mixed into a glass beaker with 100 mL of deionized water, and heated at 80 °C for 10 min. The container was sealed with parafilm to avoid water loss. After cooling, the aqueous extract was filtered via Whatman 40 filter paper and stored in amber bottles at 4 °C to avoid direct sunlight. 

### 2.3. Synthesis of Silver Nanoparticles (AgNPs)

Synthesis of AgNPs was carried out according to the methodology described in the literature with some modifications [35]. Briefly, 100 mL of *Thuja orientalis* aqueous extract was added into a 250 mL Erlenmeyer flask. The temperature of the sample was raised to 80 °C using a hot plate with a magnetic stirrer. Then, 10 mL of 20 ppm AgNO_3_ (Meyer ® A.C.S, Tláhuac, CDMX) solution was added. The green synthesis of AgNPs was monitored at six different reaction times (0, 10, 20, 30, 40, and 50 min). This time window was chosen since, after a 50 min reduction, overlapping of the surface resonance plasmon was attained. Finally, samples were allowed to cool at room temperature and stored in amber color glass bottles at 4 °C for further characterization. 

### 2.4. UV–Vis Spectroscopy

A spectrophotometer Lambda 45 UV/VIS (Perkin Elmer. Waltham, MA, USA) was used in the study of the optical properties of AgNPs. The UV–Vis spectra ranged from wavelengths of 200 to 700 nm. The absorbed energy in the UV–Vis spectra was reflected as peaks in the absorbance bands because of the AgNPs characteristic surface plasmon resonance [36]. 

### 2.5. Atomic Absorption Spectroscopy

A modified method previously described by [37] was used to detect silver ions. Briefly, the nanoparticle suspension was centrifuged (7000× *g*, 5 min) to separate AgNPs. Then, the pellet obtained was redispersed in deionized water and washed (centrifugation and redispersion) three times. Finally, the pellet was redispersed in deionized water prior to atomic absorption spectroscopy analysis. After that, 10 mL of every sample (0, 10, 20, 30, 40, and 50 min) was placed in the spectrophotometer (Perkin Elmer Atomic Analyst 200, Waltham, MA, USA). Analysis of the prepared samples took place in the equipment by the method of flame. First, atomizing the sample and then studying the absorption generated by irradiation of the atomized sample through a hollow-cathode Ag Perkin Elmer (Waltham, MA, USA), Lumina lamp (Waltham, MA, USA). A silver (Ag) standard (Merk, S.A de C.V, Naucalpan de Juarez, México) for the calibration curve was prepared in 2% HNO_3_ at concentrations of 0, 2, 4, 6, 8, and 10 mg/L. Quantification of the analyte was expressed in mg of Ag/L. 

### 2.6. Zeta Potential Analysis

Zeta potential measurements were performed using a particle size analyzer (ZetaSizer Pro, Malvern Instruments, Malvern, Worcestershire, UK). All samples were appropriately diluted with deionized water (to reduce scattering and viscosity effects) and analyzed in a disposable capillary cell DTS1070 at room temperature with an equilibration period of 120 s. The suitable parameters chosen were, for material (colloidal silver), the refractive index of 0.06 and absorption 4.276, and for dispersant (water), the refractive index of 1.33 and viscosity 0.8872 mPa/s at 25 ± 0.1 °C. Triplicates of each sample were measured, and each measurement comprised 10 runs to obtain a stable reading. Results were analyzed using the ZS Xplorer software. 

### 2.7. Transmission Electron Microscopy

The morphology of the AgNPs was examined using a transmission electron microscope (TEM; JEOL, Peabody, MAM USA) operated at an accelerating voltage of 80 kV. The high-contrast TEM is equipped with a 2k × 2k AMT CCD camera for digital image acquisition. TEM grids were prepared by placing a drop (10 µL) of the AgNPs suspension on carbon-coated grids and drying at room temperature. The micrographs of the nanoparticles were analyzed with ImageJ software version 1.8.0. Measurements were performed in approximately 500 spherical-shaped AgNPs. 

### 2.8. Preparation of films

Filmogenic solutions were prepared in a mini reactor and stirred at 125 rpm with a lightning POLYTRON System (PT2100) (Kinematica AG. Lucerne, Malters, Switzerland). Two grams of glycerol (Meyer ® A.C.S Tláhuac, CDMX) were previously diluted into 20 mL of water or colloidal solution of AgNPs and homogenized for 10 min at 60 °C. Then, 4 g of porcine skin gelatin (Gelita, Lerma, Toluca, Estado de Mexico) with 275 °B were dissolved in 40 mL of distilled water or colloidal solution at 60 °C for 10 min. This gelatin solution was added to glycerol solution by gently mixing for 10 min. Then, 180 mL of distilled water or colloidal solution was added according to the formulations illustrated in Table 1, followed by gentle mixing for 10 min at 60 °C. Subsequently, 60 g of each filmogenic solution was placed in a sterile Petri dish 23 cm in diameter. Once the solutions were cooled down at room temperature, they were dried at 25 °C in a controlled drying oven (Felisa. Zapopan, Jalisco, Mexico) for 72 h. After that, films were cut into rectangles (2 cm × 3 cm) and stored in a hermetically sealed container for 7 days at 0% RH over P_2_O_5_ (Thermo Fisher Scientific^TM^, Waltham, MA, USA). After the conditioning time, samples were ready for testing their structural and antimicrobial properties.

### 2.9. Differential Scanning Calorimetry (DSC)

The thermal stability of the films was analyzed by means of a Q2000 series DSC (TA Instruments, New Castle, DE, USA) equipped with a refrigerated cooling system (RCS90, TA Instruments, New Castle, DE, USA) and the TA2000 universal analysis software. The temperature calibration was performed with indium (melting point value of 156.6 °C), and the equipment was purged with high-purity nitrogen at a flow rate of 50 mL/min. Samples (1 ± 0.3 mg) were packed down in hermetically sealed aluminum pans (TA Instruments, New Castle, DE, USA) and scanned over the range of 20 °C to 200 °C with a heating rate of 10 °C/min. An empty aluminum tray was used as a reference. Melting temperature (Tm) and enthalpy (∆H) values were determined based on the endothermic changes recorded in the corresponding thermograms. 

### 2.10. Fourier Transform Infrared Spectroscopy

Functional groups were characterized using a Frontier FT-MIR spectrophotometer (Perkin Elmer, Waltham, MA, USA) accessorized with an attenuated total reflection (ATR) accessory. Samples were placed on the ATR diamond crystal, and the spectra were recorded in absorbance mode over the range of 4000 to 400 cm^−1^ at a resolution of 4 cm^−1^ by combining 32 scans. The background spectrum of air was subtracted from all the spectra. The main bands were analyzed using the Spectrum 10.4.2 software.

### 2.11. Antibacterial Activity

For the antibacterial activity, the plate-diffusion technique was used. The Gram-positive (*Staphylococcus aureus*) and Gram-negative (*Salmonella typhimurium*) strains were used as test pathogens. These microorganisms were inoculated into nutrients and incubated at 37 ± 2 °C for 24 h. The inoculums obtained were adjusted with respect to the McFarland equivalent of 1.5 × 10^8^ cfu/mL. Then, 0.1 mL of medium was taken with this concentration of bacteria to disperse with a swab the streaks all over the surface of the medium three different times, rotating the plastic Petri dishes (90 × 15 mm) with Mueller Hinton agar (Bioxon, Cuautitlán Izcalli, Estado de México) through an angle of 60° after each application. Finally, the swab was placed around the edge of the agar surface. The inoculum was dried at room temperature with the lid closed. Films were cut into 6 mm discs and subjected to UV light for 20 min for sterilization. Then, films were placed in Petri dishes for incubation at 37 ± 2 °C for 24 h. The inhibitory effect was determined by measuring the bacterial growth inhibition zones around the discs.

### 2.12. Color

The color of the films was determined on the CieLab scale by means of a colorimeter, model CR-400/410 (Konica Minolta, Foster City, USA). The parameters L* a* and b* were evaluated. The L* component corresponds to the luminosity. It ranges from 0 to 100 to describe the black and white color, respectively. The a* parameter describes the red (positive value) and green (negative value) colors. For the b* parameter, the positive values represent the yellow color, while negative values correspond to the blue color.

### 2.13. Experimental Design and Statistical Analysis

The experiment was conducted as a completely randomized design with three replicates. Data were analyzed by one-way analysis of variance (ANOVA), and means were separated using the Tukey test with the Minitab 16.0.1 software (Penn State University, State College, PA, USA). A significance value of α = 0.05 was used to distinguish significant differences.

## 3. Results

### 3.1. UV–Vis Spectral Analysis

**Figure 1:**
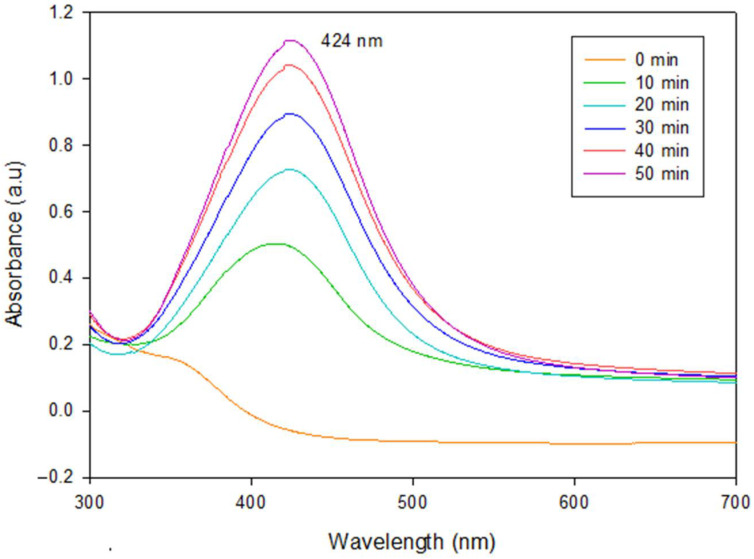
UV–Vis absorption spectra of AgNPs taken at different synthesis times.

Synthesis of AgNPs from AgNO_3_ usually involves a change in the coloration of the solution. Before synthesis, it was slightly green; however, after 50 min of synthesis, the extract shifted to an amber color which is characteristic of the AgNPs formation [38]. To corroborate the presence of AgNPs, UV–Vis spectrophotometry was performed on the aqueous solutions obtained from every 10 min of synthesis until 50 min were completed. As illustrated in Figure 1, all spectra showed bell-shaped bands as the reaction progressed. The higher the time, the greater the absorption observed at 424 nm. No absorption was detected in control samples (0 min). Furthermore, 50 min treatment exhibited the highest absorption. This value was higher compared with that reported by Liang et al. [39]. They detected a surface resonance plasmon (SRP) at around 397 nm. This difference could be attributed to the use of a different synthesis methodology (chemical reduction), where glucose acted as a reducing agent and polyvinylpyrrolidone (PVP) as a stabilizing agent. By the same route of synthesis, Kaur et al. [40] obtained an SRP around 400 nm, which could be associated with the formation of AgNPs in the presence of different agents such as trisodium-citrate and PVP. Rahman et al. [41] used the green synthesis approach by using the microalga *Chlamydomonas reinhardtii* as a reducing agent and AgNO_3_ as a precursor. They reported the SRP at 425 nm. These results agree very well with those reported in this work. The bell-shaped absorption band at a maximum wavelength of around 400 nm could be indicative of the spherical shape of the silver particles and also be associated with the reduction of Ag^+^ to Ag^0^ [42]. 

**Figure 2:**
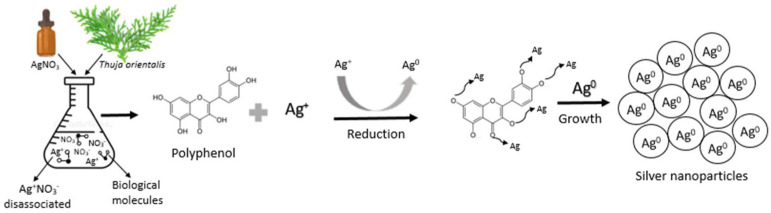
Schematic representation for green synthesis of silver nanoparticles with *Thuja orientalis*. Adapted from [3,46,47].

Plants possess a great number of secondary metabolites such as flavonoids, polyphenols, and reducing sugars. These metabolites commonly have hydroxyl groups in their structures and act as reducing/capping agents in the formation of nanoparticles [43]. Makrov et al. [44] reported the idea that flavonoids or phenolic compounds are capable of reducing metal ions by releasing a reactive hydrogen radical that can reduce the metal and thus allow the formation/stabilization of nanoparticles. The synthesis of AgNPs by reduction is carried out in several steps because of the presence of phenols in the aqueous extract. Figure 2 shows a schematic representation of the synthesis of AgNPs with *Thuja orientalis* aqueous extract. In the first step, AgNO_3_ dissociates in the aqueous solution; as a consequence, silver ions possess a partial positive charge (Ag^+^) while the nitrate ion shows a partial negative charge. Subsequently, deprotonation of phenolics occurred, generating the hydrogen radical followed by electron transfer to silver ions (Ag^+^), resulting in the formation of AgNPs. Therefore, hydroxyl-rich compounds present in the *Thuja orientalis* aqueous extract [8] can act as a reducing and stabilizing agent during the formation of AgNPs [45]. 

### 3.2. Atomic Absorption Spectroscopy

**Figure 3:**
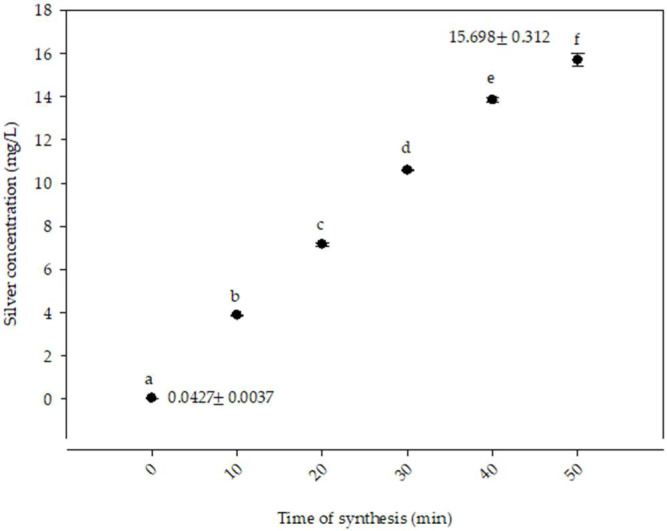
Silver ion concentration with respect to the synthesis time of AgNPs. Different letters show statistically significant differences (*p* ≤ 0.05) between treatments.

Figure 3 shows the concentration of silver ions with respect to the time of synthesis. Significant differences were recorded (*p* ≤ 0.05) among the treatments studied. At time 0 min, the lowest concentration of silver ions was reported at 0.04 ± 0.003 mg/L. The highest concentration was obtained at the 50 min treatment with 15.7 ± 0.312 mg/L. These results were higher compared with those reported by Gruszka et al. [48]. They determined the silver concentration in nanoparticles of different sizes and found a concentration of 14.5 μg Ag/L with nanoparticles of 60 nm in size. The difference in the Ag concentration could be attributed to the influence of the stabilizing agents. The results of the present research are similar to those reported by Sharma et al. [49], who synthesized AgNPs through a green route by using the extract of *Myristica fragrans* (nutmeg). In their study, a 5.5 ppm standard solution of AgNO_3_ was initially prepared. Then, the extract was added, and the concentration of Ag^+^ ion in the reaction solution was evaluated and monitored at regular intervals. The 0 min treatments reported a concentration of 5.5 ppm of Ag^+^. However, the 12 min treatment resulted in a concentration of 0.06 ppm of Ag^+,^ indicating the conversion from Ag^+^ to Ag^0^. This conversion can be attributed to the formation of enthalpies, the breaking of the chemical bonds of the AgNO_3_ elements, and the formation of new bonds to produce the silver ion Ag^0^ [50].

### 3.3. Fourier Transform Infrared Spectroscopy

**Figure 4:**
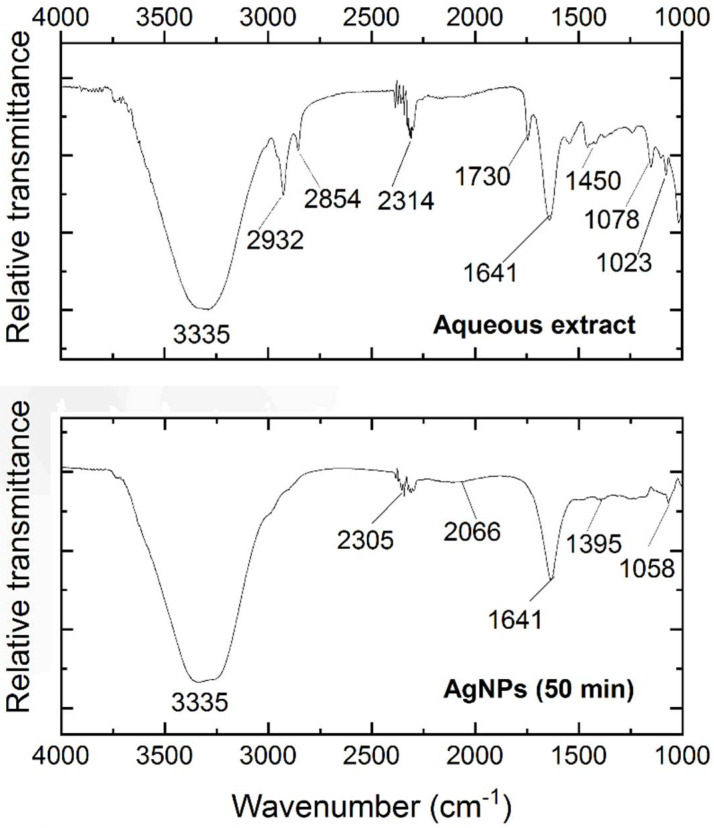
FTIR spectra of aqueous extract of *Thuja orientalis* and AgNPs obtained at 50 min of synthesis.

The FTIR spectroscopy was applied to identify the functional groups of the components present in the *Thuja orientalis* aqueous extract and a sample at 50 min of synthesis of nanoparticles. The aqueous extract showed a wide band at 3335 cm^−1^ [35] (see Figure 4). Luna-Sánchez et al. [51] reported that bands between 3709 and 2803 cm^−1^ could be related to the symmetric stretching of R–O–H. A band was observed at 2932 cm^−1,^ which could be assigned to the stretching vibrations of C–H. This band was also reported by López-Millán et al [52]. The stretching of OH groups was associated with the band at 2854 cm^−1^ [53]. At 2314 cm^−1^, a band was detected, which could be attributed to the O=C–OH [22]. Rehab-Ali et al. [54] reported similar bands at 1730 cm^−1^ and 1450 cm^−1^ associated with the C=O and C–C=C bonds, respectively. Bands between 1459 cm^−1^ and 1641 cm^−1^ have been reported by Dada et al. [55] and correlated to the C=C linkage. Both 1078 cm^−1^ and 1023 cm^−1^ bands could be assigned to the C–OH linkage and the C–O–C bending mode, respectively [52].

The spectra at 50 min of synthesis showed bands between 3709 cm^−1^ and 2803 cm^−1^ related to the symmetric stretching of R–O–H; these bands could be associated with primary or secondary amines [51]. Compared to the aqueous extract spectrum, bands with lower intensity were found at 2305 cm^−1^ attributed to the O=C group [35]. The band comprised at 2066 cm^−1^ is commonly attributed to the –CH- group [55]. Moreover, the band at 1633 cm^−1^ corresponded to the stretch of the amide C=O [56], and the band at 1395 cm^−1^ reflects the possible presence of the aromatic amine –C–N– [57]. Finally, the stretch of the –C–OH– is commonly assigned to the band at 1058 cm^−1^ [58].

### 3.4. Zeta potential Determinations

**Figure 5:**
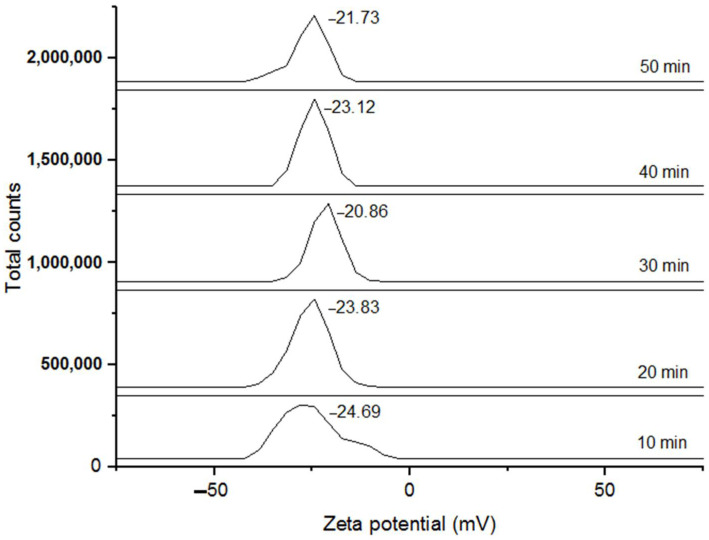
Zeta potential measurements of silver nanoparticles at different synthesis times.

Zeta potential expresses the stability of nanoformulations. Segregation and sedimentation of particles in solutions impair electrophoretic mobility. Particles below 20 nm show high mobility, low light diffusion, and a narrow concentration spectrum. Particle size influences the minimum concentration to perform the zeta potential test because it affects the particle surface charge [59]. Figure 5 shows the zeta potential analysis of the synthesized AgNPs at different times. The results varied significantly, and there was no clear tendency of increment/decrement as a function of time. 

Lunardi and coworkers [59] reported that natural organic matter stabilizes suspensions; however, if the suspension is unstable with a tendency to aggregate and polydisperse, it will be unstable over time, and the zeta potential becomes time-sensitive.

The samples 20, 30, 40, and 50 min resulted in values of −23.83, −20.86, −23.12, and −21.73 mV, respectively. These values were slightly lower than those found by Varadavenkatesan et al. [60], who synthesized nanoparticles with an extract of *Thunbergia grandiflora* and reported a zeta potential value of −24.5 mV. All these results highly agree with the present research because the zeta potential results had negative values. This could be attributed to the formation of some layers of biomolecules of the extracts that cover the nanoparticles. Additionally, the repulsive forces between the negatively charged particles could prevent coalescence or agglomeration, which could benefit the stability of the nanoparticles [61]. Previous experimental work synthesized AgNPs stabilized with cysteine with improved stability. Higher zeta potential values were reported with +44 mV at pH 4 and −52 mV at pH 9 [62]. Based on these results, the stability of the nanoparticles could be slightly improved in relation to the synthesis time. This is the reason why the 50 min treatment was selected due to its small particle size and improved zeta potential. 

### 3.5. Transmission Electron Microscopy

**Figure 6:**
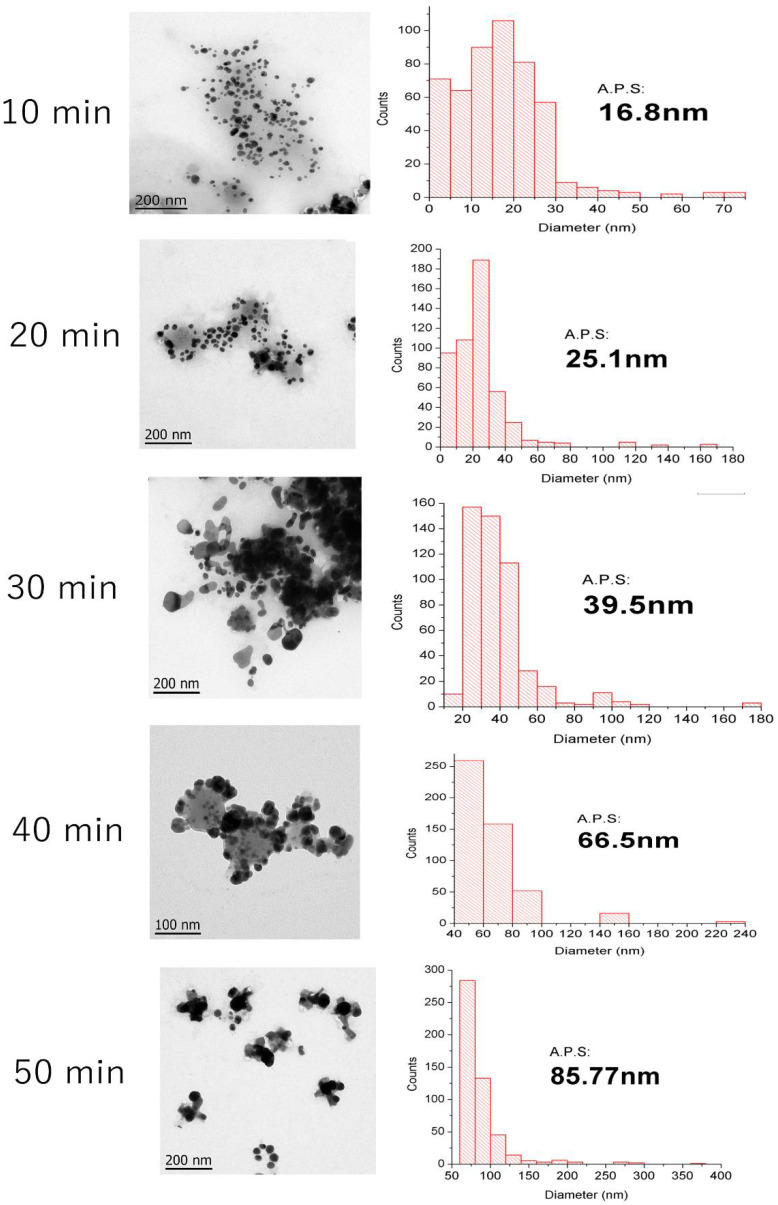
TEM images of the green synthesized AgNPs at different times. The A.P.S. means Average Particle Size.

Figure 6 elucidates the AgNPs sample sizes at different reaction times, displaying the metallic core of nanoparticles in solution through TEM images and the quantitative analysis of the particle sizes obtained at different times through histograms (500 particle counts per time). Most of the nanoparticles showed a spherical shape and indicated the successful formation of colloidal particles from silver ions in the presence of the aqueous extract. This morphology agrees with previous reports of AgNPs obtained through comparable green synthesis methods [63,64,65]. The Average Particle Size (A.P.S.) of the AgNPs varied at different reaction times, exhibiting smaller diameters (15–25 nm) when exposed to 10–20 min of reaction time. Samples synthesized at 10 min showed an increment in dispersion with slight agglomeration. However, after 20 min of synthesis, the AgNPs begin to agglomerate significantly. Samples from 50 min of synthesis resulted in a significant aspect of agglomerated AgNPs with respect to the rest of the samples. This agglomeration difficulted the measurement of size; however, the A.P.S. at 50 min was about 85.77 nm. These AgNPs showed a stretched morphology because of the aggregation of two or more AgNPs. The difference in size is commonly related to the nucleation and growth of the nanoparticles as a function of synthesis time [66]. Salayová et al. [67] reported that the different size distributions are the result of the participation of various biomolecules in the coating and bioreduction in the AgNO_3_ solution. Belteky and coworkers [68] reported the aggregation behavior of AgNPs synthesized with green tea aqueous extract. Results obtained with TEM showed a Z-average value of 87.7 nm. These results suggest that aqueous extract from *Thuja orientalis* possesses the same capacity for the reduction of Ag, yielding AgNPs with similar average particle size. 

### 3.6. Performance of Silver Nanoparticles on Gelatin Films

#### Differential Scanning Calorimetry in Films

Table 2 shows the thermal properties of gelatin films with different concentrations of AgNPs. All film samples exhibited glass transition (Tg) and melting (Tm) temperatures. Tg is the beginning of the segmental movement in a polymer from hard, glassy material to a soft, rubbery material related to crystallization and molecular mobility [69]. The G0 treatment exhibited a Tg value of 73.71 °C. This value was higher than those reported in the literature (56.4 °C) [70]. Tg decreased as the AgNPs concentration increased; the G90 and G180 treatments resulted in significantly different values to the others (*p* ≤ 0.5); they showed 66.97 °C and 66.42 °C, respectively. Similar results were obtained by Boughriba et al. [71], who mentioned that the decrease in Tg could be attributed to a state of disorder between gelatin molecules or its net structure. In addition, lower Tg values are related to the decrease in hydrogen bonds initially present in the G0 film. Gelatin is a heterogeneous mixture of single and multistranded polypeptides, each with helical proline conformations that can form films with physically cross-linked collagen structures. From 100 °C, the thermograms showed the beginning of another thermal event commonly called melting temperature (Tm). There were slight increments in Tm as the concentration of nanoparticles increased within the matrix of the gelatin film. Considering that enthalpy is the energy necessary to disorganize a molecular structure, the results obtained suggested that the presence of AgNPs increased the enthalpy of samples. For example, the treatment G180 presented a higher enthalpy value (2.084 J/g). Previous studies from Kanmani and Rhim [72] reported that the addition of AgNPs to gelatin film increased their thermal stability. This increment could be attributed to the fact that metallic silver is more heat stable. Therefore, it can be deduced that the G180 treatment resulted in better structural organization compared with the other treatments.

### 3.7. FTIR in Films

**Figure 7:**
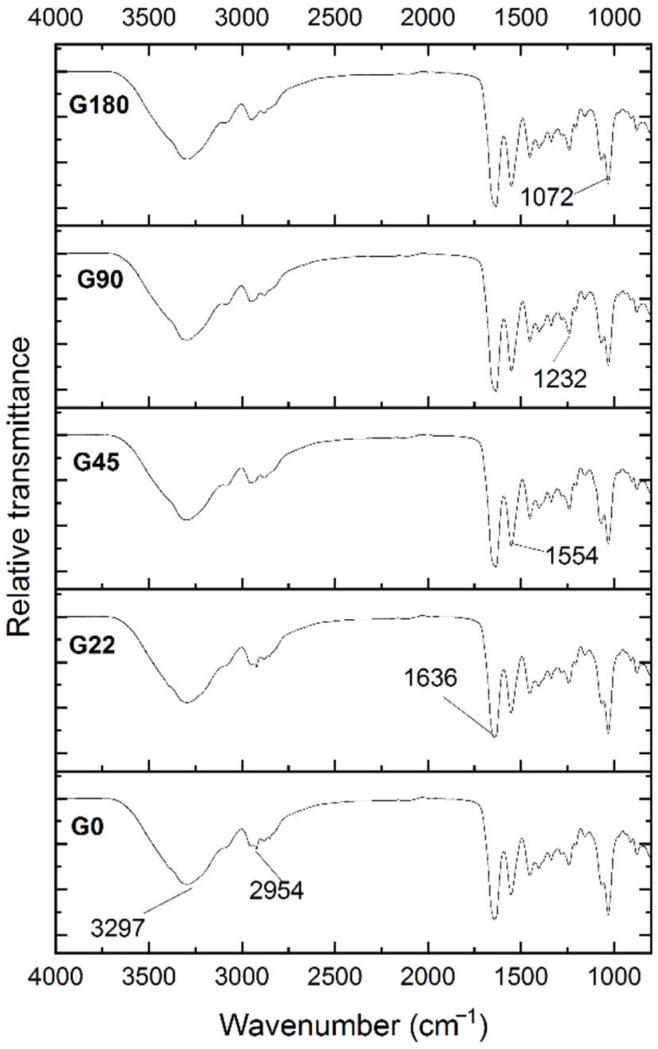
FTIR spectra of gelatin film formulations with different concentrations of silver nanoparticles.

Figure 7 shows the FTIR spectra of the gelatin films added with AgNPs. All treatments showed a similar spectral profile, in which the main bands included the amide bands A, B, I, II, and III. Bands observed in the range of 1600 to 1700 cm^−1^ correspond to amide I, which means that the C=O stretching vibration is associated with the N–H bending and stretching of the C–N [73]. Amide I in the present study was located in the 1636 cm^−1^ region, and Amide II vibration can be located at around 1500–1600 cm^−1^; the band detected at 1545 cm^−1^ could indicate the frequency of the C=O carbonyl groups stretching vibrations along the peptide bond [72]. Amide III was located at 1232 cm^−1^ because of the combination of C–N stretching and N-H deformation, which implies the complexity of intermolecular interactions as well as the relationship of the hydrogen bonds involved in the maintenance of the native structure [74]. The amide A band was located at 3297 cm^−1^ associated with the stretch vibration of the N-H group. The amide B band at 2954 cm^−1^ was assigned to the asymmetric stretch of the CH2 group [75]. The band at 1072 cm^−1^ is attributed to C-O stretching vibrations and is characteristic of the glycerol component [76]. All the infrared spectra of films presented a marked difference in magnitude. The higher the nanoparticle concentration, the higher the intensity of the bands. The absence of new bands suggests that there was no formation of a chemical bond between gelatin and nanoparticles. Moreover, the incorporation of nanoparticles did not change the structure of the gelatin film. However, the relationship between gelatin and AgNPs could be explained in terms of interactions with hydrogen bonds or van der Waals forces [77].

### 3.8. Antimicrobial Activity of Films

**Figure 8:**
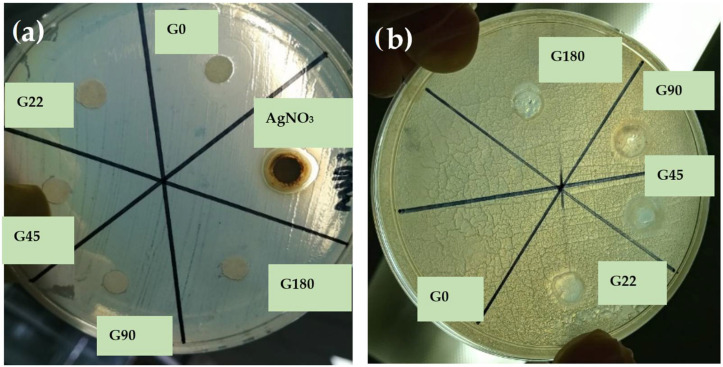
Antimicrobial effect of the films against (**a**) *Salmonella typhimurium* with no antimicrobial effect and (**b**) *Staphylococcus aureus* exhibiting inhibition halos.

The antimicrobial properties of the different film formulations with AgNPs were evaluated against Gram-negative (*Salmonella typhimurium*) and Gram-positive (*Staphylococcus aureus*) bacteria to show inhibition halos and their antimicrobial effect. From Figure 8 (profile a), the films showed no inhibition against *S. typhimurium*. According to Jamróz et al. [78], Gram-positive bacteria have a thick peptidoglycan layer of about 20–80 nm consisting of peptide-reticulated polysaccharide chains that form a complex matrix. This structure is very difficult for nanoparticles to penetrate. In contrast, Gram-negative bacteria have a ~7–8 nm peptidoglycan layer, which facilitates the entry of nanoparticles. However, penetration of AgNPs into bacteria was difficult because the lowest nanoparticle size obtained in this research was about 86.82 nm. With regard to the effects of nanoparticles on pathogens, the smaller the size of the AgNPs, the better they can adhere to the cell surface and thus spread rapidly across the membrane of the microorganisms, causing their death [79].

In general, Ag nanoparticles, silver ions, and silver nanoclusters have perfect antibacterial activity, but it is assumed that AgNPs and nanoclusters have stronger activity than silver ions since they present the greatest surface area and can easily reach the nuclear content of bacteria [80]. The antibacterial activity can be modified with the size of silver nanoparticles; its activity decreases with an increase in particle size. Regarding particle shape, truncated triangular silver nanoplates with a (111) lattice plane displayed the strongest biocidal action compared with spherical and rod-shaped nanoparticles [81]. The evaluation of the films against *S. aureus* showed good inhibition halos compared with the control treatment (G0), as shown in Figure 8 (profile b). The lower the concentration of nanoparticles, the higher the inhibition halo. Treatments with higher concentrations of AgNPs exerted less inhibitory effect, and it can be attributed to the fact that nanoparticles tend to agglomerate in the absence of any surfactant [79]. There are two ways to control bacterial growth in antimicrobial action. Firstly, there is a modification of the stabilization of the nanoparticle surface according to the microbial environment through the processes of dissolution, aggregation, photo reductive reaction, and release of Ag^+^ ions from the AgNPs. Secondly, the interaction of Ag^+^ ions with bacterial cell walls results in the inhibition of bacterial protein functions, stopping bacterial growth and consequently causing their death. AgNPs adhere and accumulate on the surface of bacteria altering membrane properties. Once the AgNPs have penetrated the membrane, they release silver ions that can interact with thiol-containing proteins in the cell wall and inhibit bacterial protein functions, which stops bacterial growth and leads to bacterial death [82]. Arfat et al. [83] reported that the release of silver ions might penetrate through voids or perforations in the bacterial membrane. Nanoparticles may interact with disulfide or sulfhydryl groups that possibly adhere to the negatively charged cell wall, resulting in disruption of metabolic processes or rupture of the bacterial cell wall causing cell death. Based on these results, G22 can be considered the best treatment because of the effectiveness of nanoparticles; it contained a lower concentration of AgNPs but higher antimicrobial activity. These results suggested that gelatin solutions with a higher concentration of nanoparticles considerably affected the agglomeration of nanoparticles, increasing their size. It resulted in difficulty in penetrating the cell wall of the microorganisms and, therefore, reducing their antimicrobial activity.

### 3.9. Color of The Films

**Figure 9:**
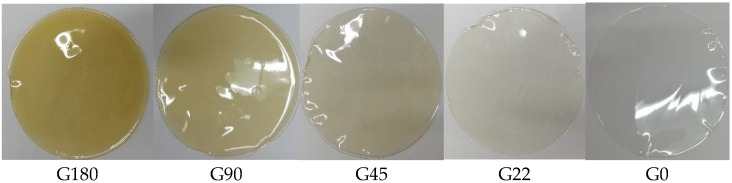
The color difference in the film formulations with respect to the concentration of silver nanoparticles.

The color of gelatin films added with different concentrations of AgNPs is shown in Table 3. As the concentration of nanoparticles decreased, the a* and b* coordinates decreased equally. However, the L* parameter decreased significantly (*p* ≤ 0.05) toward darker color as the concentration of nanoparticles increased. The control sample (G0) showed an L* value of 91.87; however, the sample with the higher concentration of nanoparticles (G180) resulted in L* values of about 85.38. In this context, Pathare et al. [84] reported that the parameter a* produces positive values for reddish colors and negative values for greenish ones, while b* takes positive values for yellowish colors and negative values for blue ones. L* is an approximate value of brightness between black and white. The parameter a* yielded values from −0.206 to 1.223 and showed a tendency towards reddish colors as the concentration of nanoparticles increased. Regarding the parameter b*, its values increased from 1.883 to 17.1, showing a preference for yellow colors. Previous results by Jamróz et al. [78] reported that increments in the concentration of AgNPs in gelatin films produce an increase in total color difference. These numerical results can be observed as images in Figure 9. It shows the color changes; the G0 treatment was visually transparent to translucent. However, as the concentration of AgNPs increased, the films showed a yellowish color. The G180 treatment showed a more intense yellow color close to amber.

## 4. Conclusions

The aqueous extract of *Thuja orientalis* is an effective reducing/capping agent for the synthesis of AgNPs at 50 min. There was evidence of the formation of nanometric silver particles with an average particle size of about 85.77 nm. This methodology represents an environmentally friendly alternative for the synthesis of AgNPs. The films added with AgNPs had a significant inhibitory effect on the growth of *Staphylococcus aureus*. Films from G22 could serve as a possible substitute for commercial packaging materials with improved antimicrobial properties. Gelatin films are biodegradable materials and help reduce environmental pollution. They can inhibit the growth of microorganisms and therefore increase the shelf life of food. These biomaterials could be adapted to the needs or characteristics necessary in the food and biomedical industries. 

## Figures and Tables

**Table 1:** The composition used in the preparation of gelatin films.

Key	Distilled Water (mL)	Colloidal Solution (AgNPs)(mL)	Gelatin (g)	Glycerol (mL)
G180	0	180	4	2
G90	90	90	4	2
G45	135	45	4	2
G22	157.5	22.5	4	2
G0	180	0	4	2

**Table 2:** Thermal properties of gelatin films with different concentrations of silver nanoparticles. Different letters represent significant differences (*p* < 0.05).

Treatments	Tg (°C)	Tm (°C)	∆H (J/g)
G0	73.71 ± 0.531 ^a^	102.93 ± 0.132 ^ab^	1.731 ± 0.754 ^ab^
G22	73.66 ± 0.316 ^a^	102.40 ± 0.648 ^b^	2.227± 0.548 ^a^
G45	72.41 ± 0.786 ^a^	103.09 ± 0.188 ^ab^	1.821 ± 0.014 ^ab^
G90	66.97 ± 0.347 ^b^	103.17 ± 0.030 ^ab^	1.340 ± 0.028 ^b^
G180	66.42 ± 0.565 ^b^	103.31 ± 0.179 ^a^	2.084 ± 0.110 ^b^

**Table 3:** CIELab color parameters obtained in the gelatin-based films. Different letters represent significant differences (*p* < 0.05).

Treatments	L*	a*	b*
G0	91.87 ± 0.182 ^a^	−0.206 ± 0.011 ^e^	1.883 ± 0.005 ^e^
G22	90.683 ± 0.073 ^b^	−0.613 ± 0.005 ^d^	4.27 ± 0 ^d^
G45	90.49 ± 0 ^c^	−0.666 ± 0.011 ^c^	5.066 ± 0.005 ^c^
G90	88.276 ± 0.153 ^d^	0.14 ± 0 ^b^	9.68 ± 0.01 ^b^
G180	85.38 ± 0.199 ^e^	1.223 ± 0.015 ^a^	17.1 ± 0.051 ^a^

## Abbreviations

AgNPs	Silver Nanoparticles
SPR	Surface Plasmon Resonance
FTIR	Fourier transform infrared spectroscopy
